# From silos to synergy: the interdisciplinary discovery potential in redefining polyendocrine metabolic ovarian syndrome

**DOI:** 10.1172/JCI209199

**Published:** 2026-09-15

**Authors:** Michaela M. Morhaus, Lauren W. Yowelunh McLester-Davis, Judith A. Simcox

**Affiliations:** 1 Department of Biochemistry and; 2 Cell Molecular Biology Program, University of Wisconsin–Madison, Madison, Wisconsin, USA.; 3 Medical Scientist Training Program, University of Wisconsin School of Medicine and Public Health, Madison, Wisconsin, USA.; 4 Howard Hughes Medical Institute, Department of Biochemistry, University of Wisconsin–Madison, Madison, Wisconsin, USA.

## Abstract

Polyendocrine metabolic ovarian syndrome (PMOS) affects one of every eight women worldwide and is associated with high rates of cardiovascular disease, type 2 diabetes, and infertility. Polycystic ovarian syndrome (PCOS), another term for this condition, reflects a historical focus on its effects on the female reproductive system. Recently, however, experts and patient groups have advocated renaming the condition PMOS, conveying increasing recognition that hyperandrogenism and insulin resistance contribute to its pathology. In this issue, Nguyen et al. took a systems genetic approach that combined data from murine models of PMOS and data from human studies, identifying the splicing factor 3b subunit 1 (SF3B1) and IGFBP2, both previously associated with metabolic disease, as molecular drivers of PMOS. SF3B1 inhibitors improved PMOS symptoms in mice, including decreased adiposity, hypoandrogenism, and insulin levels. These findings open therapeutic and mechanistic avenues in the study of PMOS, including exploration of splice variants as regulators of disease progression.

## Introducing PMOS: recharacterizing PCOS through its metabolic features

Polycystic ovarian syndrome (PCOS) was first described in 1935 by gynecologists Stein and Leventhal, with their report linking polycystic ovarian morphology, clinical hyperandrogenism, and oligomenorrhea (1, 2). This name placed the condition firmly under the purview of gynecology — a surgical specialty focused on female reproductive anatomy and physiology. This gynecological characterization of PCOS persisted despite growing recognition that hyperandrogenism and insulin resistance are the core components of the pathophysiology, while the ovulatory dysfunction, including cystic ovaries, represented a downstream effect. The recent push to rename PCOS as polyendocrine metabolic ovarian syndrome (PMOS) represents a long-overdue update that more accurately reflects modern understanding of its pathophysiology (3). PMOS emphasizes the association with other metabolic diseases, including cancer, cardiovascular disease, type 2 diabetes, and insulin resistance, the latter of which is found in 50%–80% of patients with PMOS (4). The reclassification of PMOS carries the aspiration that research and care teams will evolve to include interdisciplinary collaborations that span reproduction, endocrinology, and metabolic aspects of the disease.

The work of Nguyen et al. presented in this issue of the *JCI* represents a major advance in interdisciplinary collaboration through its systems genetic approach to identify molecular drivers of cardiometabolic disease in PMOS (5). The study integrated murine genetic, molecular, and phenotypic data to identify mechanisms that control metabolic health and cross-referenced these findings with human clinical and molecular profiling datasets. PMOS in mice was induced through the use of an aromatase inhibitor, which increased follicle number, ovarian volume, adiposity, insulin resistance, and hyperandrogenism. Using various mouse strains, Nguyen et al. were able to model PMOS subtypes A–D, which range in hormone and metabolic profiles (6). These PMOS subtypes also have distinct treatment responses, with subtype A being more responsive to antiandrogen therapy and hormonal birth control, while subtype D treatments focus on interventions for fertility, such as ovulation induction (7). Refining disease models to capture previously unrecognized subtypes creates a pathway for personalized, mechanism-based interventions, moving beyond generalized treatments for symptom management.

## SF3B1 and IGFBP2 emerge as drivers of PMOS

Two important mechanistic findings emerged from the study: that the metabolic underpinnings of PMOS are regulated by splicing factor 3b subunit 1 (SF3B1) and that ovarian-adipose crosstalk is regulated by insulin-like growth factor binding protein 2 (IGFBP2). Both SF3B1 and IGFBP2 are known regulators of metabolic disease that toggle the molecular switch driving cancer proliferation and insulin resistance, respectively.

**Figure 1:**
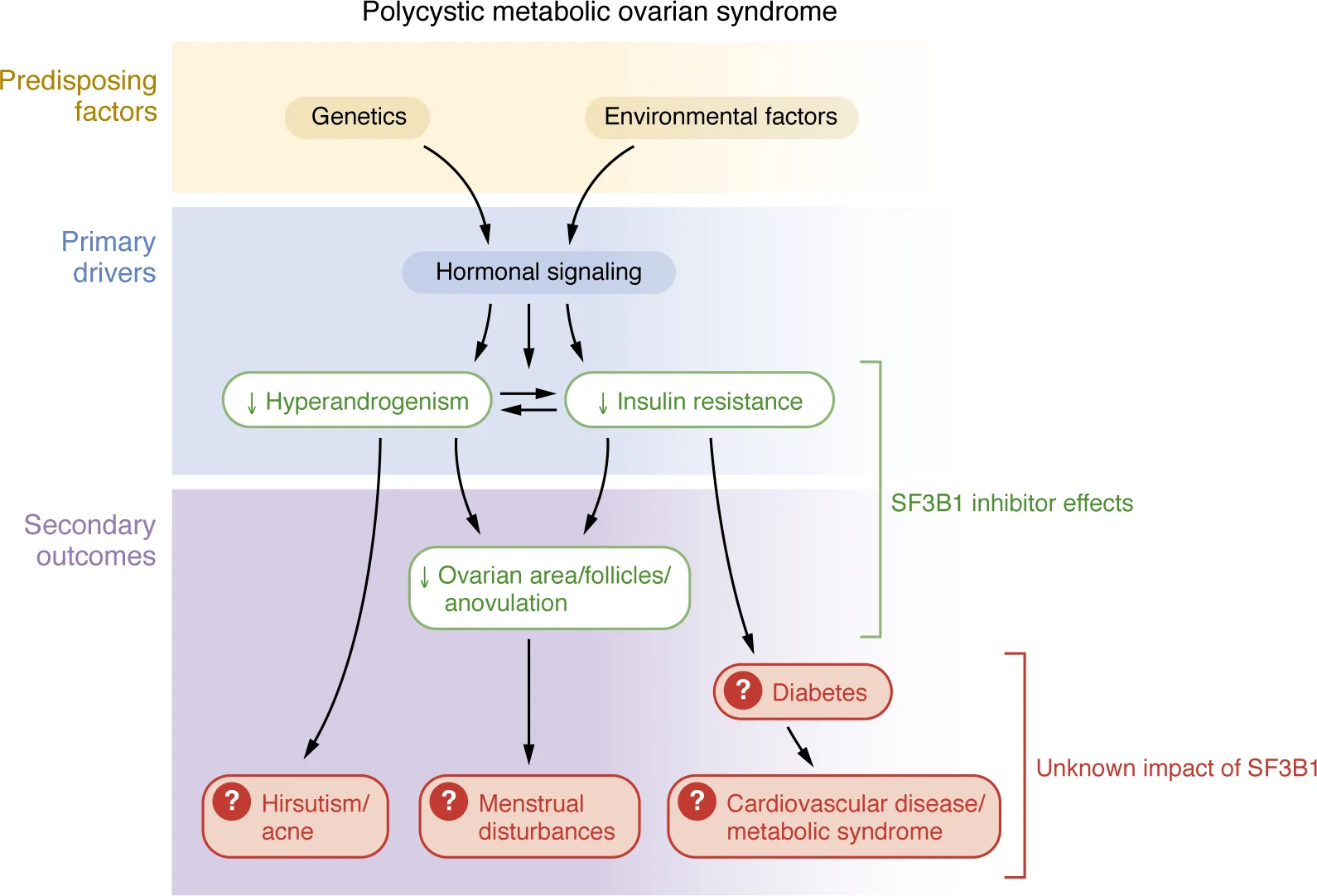
SF3B1 inhibitors improve primary drivers of PMOS. The study by Nguyen et al. (5) utilized mouse models to understand the contribution of PMOS predisposing factors (yellow box) as well as the phenotypic range of primary drivers (blue box) and secondary outcomes (purple box). One factor that emerged from the investigation was splicing factor 3b subunit 1 (SF3B1). Treatment of PMOS mouse models with SF3B1 inhibitor decreased primary drivers of PMOS, including hyperandrogenism and insulin resistance, as well as consequent outcomes, including those that affect the ovarian area (green text). More research is needed to understand the effect of SF3B1 inhibitors on other secondary outcomes of PMOS, including hirsutism, diabetes, menstruation, and cardiometabolic disease (red text).

SF3B1 is a part of the U2 small nuclear ribonucleoprotein complex of spliceosomes. Cancer-associated mutations in SF3B1 decrease mitochondrial respiration through missplicing of metabolic enzymes, including ubiquinol-cytochrome *c* reductase complex assembly factor 1 and methylmalonyl-CoA mutase (8). In SF3B1 mutation-driven cancers, treatment with SF3B1 inhibitors decreased proliferation (9). Employing these SF3B1 inhibitors in mouse models of PMOS decreased fat mass, hyperandrogenism, and insulin levels in a PMOS subtype-specific response (5) (Figure 1). These exciting results target primary drivers of PMOS. Further work is needed to understand the effect of SF3B1 inhibitors on secondary disease outcomes; this work will require innovative tools, such as the use of menstruating mouse models (10). Additionally, the mechanistic role of SF3B1 in PMOS remains unknown but may control specific missplicing events to regulate metabolism and ovarian morphology. The tissue- and cell-specific impact of SF3B1 inhibition also warrants further investigation to elucidate off-target effects, which will be needed for translational studies to uncover if these SF3B1 inhibitors can be employed to treat PMOS in humans.

The second mechanistic finding of the study identified IGFBP2 as a regulator of ovarian-adipose crosstalk, which aligns with observations that IGFBP2 controls adiposity and adipokine production. Human studies have shown that serum IGFBP2 decreases in type 2 diabetes, insulin resistance, and obesity. Overexpression of IGFBP2 in insulin-resistant mouse models improved insulin sensitivity and hepatic steatosis and decreased visceral adipose tissue depots (11). Further exploration of IGFBP2 as a domain-specific peptide therapy could be warranted in PMOS, given that IGFBP2 heparin-binding domain 1 and 2 peptide therapy decreases adiposity in mouse models of obesity by 48% (12).

## Looking ahead: advancing big data and interdisciplinary efforts

Nguyen et al.’s studies also highlight 2 emerging topics in human health repositories: populations surveyed and sequencing depth (5). Large data repositories with genomic and phenotypic data create inroads to translational research, including those used in Nguyen et al.’s work: the Atherosclerosis Risk in Communities (ARIC) and the Genotype-Tissue Expression (GTEx) Portal. While the innovation potential in these repositories is clear, the lack of representation and inclusion from underserved groups in biomedical research, such as Indigenous populations, leaves major gaps in discovery potential and subsequent treatments that need to be thoughtfully bridged through community engagement (13, 14). The second emerging trend is the importance of splice variants and their regulation as drivers of metabolic disease. Splice variants expand proteomic diversity, provide physiological specialization, and regulate fine-tuning through noncoding RNA regulation (15). To broaden utilization for splice variant analysis, appropriate sequencing depth will need to be standardized across repositories.

The next frontier is for interdisciplinary research collaborations to harness these discoveries to fine-tune PMOS diagnosis and medical intervention. The standard of care for PMOS has evolved with the disease name. Early surgical approaches such as ovarian wedge resection have been replaced by current recommendations that include lifestyle modification and medical weight management, alongside pharmaceutical interventions targeting multiple facets of the disorder, including insulin resistance, oligomenorrhea, and androgen excess. The name change was catalyzed by expanded knowledge of PMOS disease subtypes, the interplay of metabolic signaling, and multiple organ systems that drive the range of phenotypes. Despite these advances in treatment and research on PMOS, diagnosis remains a major challenge, with an estimated 70% of affected individuals being undiagnosed (16). Although the goals of renaming are lofty, within the last several years nonalcoholic fatty liver disease (NAFLD) was renamed metabolic dysfunction–associated steatotic liver disease (MASLD) with the intention of reducing stigma and facilitating engagement between multiple medical specialties in the care of these patients. Future emphasis of PMOS should likewise utilize molecular discoveries for diagnosis and personalized treatment strategies with coordination across care teams and medical disciplines.

## Conflict of interest

LWYMD receives income from the Robert Wood Johnson Foundation through the University of California San Francisco for grant reviewing. JAS is an author for Lehninger Biochemistry: Core Concepts and Applications and receives income from MacMillan Publishers.

## Funding support

This work is the result of NIH funding, in whole or in part, and is subject to the NIH Public Access Policy. Through acceptance of this federal funding, the NIH has been given the right to make the work publicly available in PubMed Central.

- Glenn Foundation (to JAS).
- American Federation for Aging Research (A22068 to JAS).
- Breakthrough T1D (JDRF201309442 to JAS).
- NIH/National Institute of Diabetes and Digestive and Kidney Diseases (NIDDK) grant (R01DK133479 to JAS).
- Medical Scientist Training Program (MSTP) T32 grant (5T32GM140935 to MMM).
- NIH grants (R01 AG054059, R01 AG062307, R01 AG074231 to LWYMD).
- NIDDK grant (R01DK137976 to JAS)
- JAS is an Howard Hughes Medical Institute Freeman Hrabowski Scholar.

## Version 1 09/15/2026

Electronic publication

## References

[1] Balen A (2004). The pathophysiology of polycystic ovary syndrome: trying to understand PCOS and its endocrinology. Best Pract Res Clin Obstet Gynaecol.

[2] Azziz R, Adashi EY (2016). Stein and Leventhal: 80 years on. Am J Obstet Gynecol.

[3] Teede HJ (2026). Polyendocrine metabolic ovarian syndrome, the new name for polycystic ovary syndrome: a multistep global consensus process. Lancet.

[4] Amisi CA (2022). Markers of insulin resistance in polycystic ovary syndrome women: an update. World J Diabetes.

[5] Nguyen CM (2026). Systems genetics approaches model the heritable architecture of polyendocrine metabolic ovarian syndrome. J Clin Invest.

[6] Rotterdam ESHRE/ASRM-Sponsored PCOS Consensus Workshop Group (2004). Revised 2003 consensus on diagnostic criteria and long-term health risks related to polycystic ovary syndrome (PCOS). Hum Reprod.

[7] Azziz R (2006). Controversy in clinical endocrinology: diagnosis of polycystic ovarian syndrome: the Rotterdam criteria are premature. J Clin Endocrinol Metab.

[8] Dalton WB (2019). Hotspot SF3B1 mutations induce metabolic reprogramming and vulnerability to serine deprivation. J Clin Invest.

[9] Wang S (2023). Inhibition of SF3B1 improves the immune microenvironment through pyroptosis and synergizes with αPDL1 in ovarian cancer. Cell Death Dis.

[11] Hedbacker K (2010). Antidiabetic effects of IGFBP2, a leptin-regulated gene. Cell Metab.

[12] Xi G (2013). The heparin-binding domains of IGFBP-2 mediate its inhibitory effect on preadipocyte differentiation and fat development in male mice. Endocrinology.

[13] Claw KG (2018). A framework for enhancing ethical genomic research with Indigenous communities. Nat Commun.

[14] Halmai NB (2025). Indigenous data sovereignty in genomics and human genetics: genomic equity and justice for indigenous peoples. Annu Rev Genomics Hum Genet.

[15] Tao Y (2024). Alternative splicing and related RNA binding proteins in human health and disease. Signal Transduct Target Ther.

[16] Gibson-Helm M (2017). Delayed diagnosis and a lack of information associated with dissatisfaction in women with polycystic ovary syndrome. J Clin Endocrinol Metab.

